# Additional Cases, from a Paper on the “Use of Inhalations in the Treatment of Inflammations of the Nasal Passages and Their Appendages”

**Published:** 1861-11

**Authors:** Frank W. Reilly


NOTE.—At the October meeting of the Chicago Medical Society, Dr. REILLY read a paper on the *Use of Inhalations in the Treatment of Inflammations of the Nasal Passages and their Appendages*,—which paper was ordered to be published. The foregoing, however, covering the same ground, in the main, it is not deemed necessary to do more than give one or two illustrative cases from the paper, in addition:

During the early part of May last, while visiting a patient in the North Division of the city, I was consulted by a brother of the patient, with reference to a case of chronic catarrhal cephalalgia of some eighteen months' standing, the history and symptoms of which are as follows: the patient is a man of about forty years of age, married, of active business habits, strumous diathesis. Had been under treatment about seven years at the East and here, for some disease of which I got but a vague and unsatisfactory idea. About eighteen months since, he was seized with a heavy, dull pain between and over the eyes, in the region of the frontal sinuses, gradually extending upwards, in the median line, to the vertex; this pain was aggravated without any apparent cause; was accompanied by temporary partial blindness, unsteadiness of movement and giddiness, suffusion and congestion of the eyes, with a copious milky secretion, soon becoming viscid, and obstructing vision; the sense of fulness in the frontal region was intensely painful, and easily excited by rapid movements, coughing, sneezing and loud talking. Usually these symptoms gradually disappeared, without further noticeable occurrence; but, occasionally, relief was obtained by the sudden giving way as of a sac, and the copious discharge of a thin, clear fluid, not acrid, but sometimes very offensive as to its odor. Such rupture was usually caused by some muscular straining, while bending down or at stool, and the discharge was commonly quite forcible, the fluid gushing from the nostrils in a stream. Accompanying these phenomena, at one time, was partial deafness in the left ear, resulting apparently, from an inflammation of the membrane of the eustachian tube, and relieved by a copious puriform discharge from the ear.

At the time I saw the gentleman, he was unable to raise his voice above the ordinary conversational tone, or to sneeze or cough; walked with much deliberation, being cautious about jarring himself by sudden motion. His eyes were suffused, and nose, at the base and root, red and congested in appearance. He had been treated in a variety of ways; stimulating diaphoretics, mercurial alteratives, and general antiphlogistics had each been tried, and all without relief.

Judging this to be a case of morbid condition of the mucous membrane, due to some specific irritation, by which the function of the membrane had become perverted, while its sensibility was increased, it seemed to me that local medication was desirable, and that little was to be expected, as, indeed, had been already proved, by general treatment. But little reliance, I was aware, was placed on inhalations, and yet I knew of no more efficient way of reaching the seat of the disease, namely: the schneiderian membrane, and mucous membrane of the ethmoidal cells and frontal sinuses; and aware of the success achieved by one or two members of the profession in long-standing troublesome cases of bronchial catarrh, by the use of inhalations of chloroform and iodine, I determined to make the trial. Twenty grains of iodine were dissolved in an ounce of chloroform, and the vapor inhaled slowly and steadily through each nostril alternately, ceasing when the anæsthetic effect of the chloroform became perceptible; and this was persisted in, night and morning for some six weeks. At the end of that time the inhalation was discontinued, and when about a month since, I met Mr. D., in the Board of Trade, of which he is a member, he was able to cough, sneeze and halloo as vehemently, to jump and run as actively as ever he was. The cure, however, is not a complete one; for he informs me, since, that after any unusual mental excitement,—a more than ordinarily busy day, or a too close application to business, and occasionally, without any such cause, he feels the dull, heavy pain between the eyes, premonitory of an attack; but he instantly has recourse to his vial of iodized chloroform, by which, in the course of the day, he is entirely relieved of the threatening symptoms.

Case II. is even more marked in its prompt relief. R. C.; ætat forty-five; American; occupation, superintendent of a large manufacturing establishment;—came to me, August 15th, 1861, complaining of fœtid discharge from left nostril, and dull, painful sense of fulness at the root of the nose extending to the inner canthus of the left eye; sense of fulness more marked at sometimes than others; "takes cold" easily, sneezes and "nose runs" from slight exposure ;—followed a severe cold contracted about two years before. On examination found mucous membrane of both nostrils reddened, and a slight touch with the probe in the left one, very painful, exciting the lachrymal secretion profusely, and causing violent sneezing. Ordered the iodine and chloroform, grs.xx—℥j. Saw the patient August 22d ; inflamed condition of membrane subsiding, discharge very much less ; passed curved probe up freely, merely causing tears to start, and some little efforts to sneeze afterwards, but very little, if any, pain ; pressure at base of nose, borne well. Inhalations continued. September 5th ; he had not used the iodine for four or five days. Felt completely well. Have met the gentleman within ten days, and thinks himself permanently cured.

Some five or six years since, Dr. Davis commenced the use of iodine in this manner in tuberculosis, accompanied by harrassing cough, in tubercular laryngitis, and in inflammations involving the fauces and schneiderian membrane, and with a good degree of success. I do not know that its use has been made known to the profession, nor, so far as I can learn, is it prescribed by any physician of this city, except Drs. Davis and Byford. The latter gentleman has used it quite frequently, I believe, and very successfully in the chronic catarrhs. Its ease of application, by which the costly and complicated inhaling apparatus is dispensed with, its grateful effects, and, so far, its success would seem to warrant a more thorough and extensive trial than it has, as yet, received. F. W. R.

